# The Grass Carp Genome Database (GCGD): an online platform for genome features and annotations

**DOI:** 10.1093/database/bax051

**Published:** 2017-07-27

**Authors:** Yaxin Chen, Mijuan Shi, Wanting Zhang, Yingyin Cheng, Yaping Wang, Xiao-Qin Xia

**Affiliations:** 1 Institute of Hydrobiology, The Chinese Academy of Sciences, Wuhan 430072, China and; 2 University of Chinese Academy of Sciences, Beijing, China

## Abstract

As one of the four major Chinese carps of important economic value, the grass carp (*Ctenopharyngodon idellus*) has attracted increasing attention from the scientific community. Recently, the draft genome has been released as a milestone in research of grass carp. In order to facilitate the utilization of these genome data, we developed the grass carp genome database (GCGD). GCGD provides visual presentation of the grass carp genome along with annotations and amino acid sequences of predicted protein-coding genes. Other related genetic and genomic data available in this database include the genetic linkage maps, microsatellite genetic markers (i.e. Short Sequence Repeats, SSRs), and some selected transcriptomic datasets. A series of tools have been integrated into GCGD for visualization, analysis and retrieval of data, e.g. JBrowse for navigation of genome annotations, BLAST for sequence alignment, EC2KEGG for comparison of metabolic pathways, IDConvert for conversion of terms across databases and ReadContigs for extraction of sequences from the grass carp genome.

**Database URL:** http://bioinfo.ihb.ac.cn/gcgd

## Introduction

As the only species of the genus *Ctenopharyngodon*, the family *Cyprinidae*, the grass carp (*Ctenopharyngodon idellus*) is a herbivorous freshwater fish distributed across a wide native range from the catchment area of the Pearl River in southern China to the Heilongjiang River in northeastern China (1). Since 1960 s, grass carp has been deliberately introduced into >100 countries for aquaculture and vegetation control purposes (2). Well-known as one of the four major Chinese carps, it is the species of fish with the largest production in aquaculture globally, accounting for 15.6% of global freshwater aquaculture production in 2011 (3).

The grass carp has been well studied on many macroscopic aspects: cultivation, artificial reproduction, feeding and nutrition, disease prevention and control and even aquatic weed control (4). However, research at the molecular level has been confined only to genes involved in the immune response system (5, 6), nutrition and growth (7, 8) and control of food intake (9, 10). Very little is known about the underlying molecular mechanisms of key physiological behaviors that could have economic significance. Such mechanisms might determine the long sexual maturity period of the grass carp, which extends up to 5 years and its capability to absorb nutrients from oligotrophic weeds. Usually such complex traits are determined by multiple genes in an interaction network, requiring to be investigated at the whole-genome scale.

With the rapid development of sequencing technology, the genomes of an increasing number of teleosts have been sequenced including zebrafish (11), common carp (12), medaka (13), rainbow trout (14), Atlantic salmon (15) and large yellow croaker (16) from the *Sciaenidae* family. Correspondingly, web-based databases for visualization and utilization of these genomes have been developed. Well-known databases include ZFIN (17, 18) for zebrafish, CarpBase (http://www.carpbase.org) for common carp, SalmonBase (http://salmobase.org) for the Atlantic salmon and SalmonDB for salmonids species (19).

The draft genome of the grass carp was completed in 2015 by Wang *et al.* (20). Based on this draft genome, in-depth investigations were conducted on the evolution, vegetarian adaptation, sex determination and sex differentiation of the grass carp. Undoubtedly, it is also valuable for identification of genes related to important economic traits and for selection of new varieties with advantageous traits. In brief, whole-genome sequencing opened a new era for molecular breeding of grass carp.

In order to facilitate the utilization of the draft genome and other omics data associated with the grass carp, we present the Grass Carp Genome Database (GCGD) with a web interface which allows researchers to search or to analyze genomic and transcriptomic data. GCGD comprises the genomic sequences, the genetic linkage maps, the microsatellite markers (i.e. Simple Sequence Repeats, SSR), three transcriptomic datasets and the functional annotations of 32 811 predicted protein-coding genes. The functional annotations come from successful mapping to other public databases or terms including Gene Ontology (GO), Enzyme Commission (EC) number, KEGG Orthology (KO), KEGG pathway map, etc. A series of tools have been embedded in GCGD to improve the visualization or to provide other practical functions, as examples, JBrowse (21) for browse of genomic data, BLAST (22) for sequence similarity alignment, EC2KEGG (23) for metabolic pathway comparison between two species, IDConvert for ID conversion across databases and ReadContigs for sequence extraction from the genome. As a scalable and flexible database, the genome annotations in GCGD will be constantly updated. Furthermore, other data, including multi-omics datasets, single-nucleotide polymorphisms (SNPs) and genome-scale metabolic networks, are supposed to be introduced into the database gradually in the future.

## Data analysis and preparation

### Gene nomenclature

Following the 27 263 genes originally reported (20), 5548 additional genes have been predicted by the authors (unpublished), so the total gene number reaches 32 811. To name these predicted protein-coding genes of grass carp, all predicted protein sequences were aligned with sequences in Swiss-Prot and ZFIN database using BLASTP by a threshold of e < 1e-6 and score > 100. For each alignment, corresponding gene in GCGD was named as the aligned protein in Swiss-Prot or ZFIN. For example, the gene ID ‘CI_GC_3021’ was named ‘cyclin-G2 [Danio rerio]’. Other genes failed in aligning were uniformly named as the prefix ‘_PREDICTED:gene:’ followed by their locations on the draft genome. For example, the gene with ID ‘CI_GC_80’ is named as ‘_PREDICTED:gene:CI01000000_02237986_02243555’. A total of 28 771 out of 32 811 predicted protein-coding genes have been named as their counterparts in Swiss-Prot or ZFIN (Table 1).

**Table 1.** Summary of the functional annotations of 32 811 predicted protein-coding genes

Annotation type	No. of genes
Swiss-Prot or ZFIN	28 771
Protein term/ID	32 811
GO (Gene Ontology terms)	19 144
KO (KEGG orthology)	5632
EC (Enzyme commission number)	4312
KEGG pathways	5632
Gene family (TreeFam)	23 842
Collinear gene with zebrafish	21 392
Single-copy orthologous genes with zebrafish	11 404

### Gene collinearity and genetic linkage map

The gene collinearity between the grass carp and the zebrafish (*Danio rerio*, genome version Zv9) was analyzed by Wang (20), and Circos (version 0.68-1) (24) was used to visualize it. The zebrafish genome is composed of 25 pairs of chromosomes (11) and the grass carp has 24 pairs. However, the draft genome of the grass carp has not yet been assembled to the level of chromosomes, and consequently the top 40 of 164 386 scaffolds, which covered the most number of collinear genes with zebrafish, were selected for this visualization. Based on a previously published linkage map of the grass carp (25), a consensus linkage map was reproduced for searching and browsing in GCGD. Marker names and genetic distances of 16 SNPs and 263 SSRs were collected and mapped to 24 linkage groups (LGs) using MapChart (26).

### Functional annotations

To obtain the functional annotations of 32 811 predicted protein-coding genes, we applied a series of software tools and databases including BLASTP, Blast2GO (27), Gene Ontology (28), KEGG (29), BRENDA (30). The annotations were listed as name/alias, GO, KO, KEGG pathway map and EC number, etc. (Table 1). >20 000 genes were clustered into gene families by Treefam (31) (*P *<* *le-10 and score  > = 100). Based on a list of EC numbers, 130 KEGG pathway maps were found by a comparative analysis with EC2KEGG. These maps could display the distinctions of metabolic pathways between the grass carp and the zebrafish.

### SSRs

**Figure 1.**
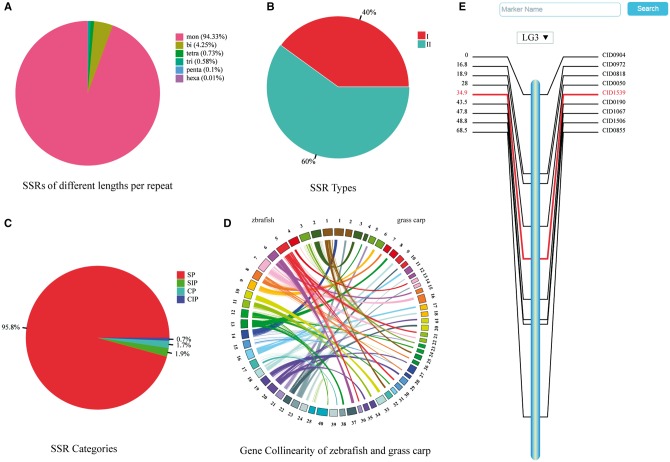
Summaries of SSRs, gene collinearity map, and a consensus linkage map. (**A**) Percentage of SSRs of various lengths per repeat. (**B**) Percentage of two types of SSRs. (**C**) Percentage of four categories of SSRs. (**D**) Gene collinearity between zebrafish and grass carp. Colored arcs connect homologous genes in zebrafish (left) and grass carp (right). (**E**) Genetic map of the third linkage group.

An in-house python script was used for genome-wide scanning, identifying >6 947 000 SSRs. The microsatellites were counted based on the number of nucleotides per repeat: mononucleotide (6 866 782, 94.33%), dinucleotide (309 157, 4.25%), trinucleotide (42 533, 0.73%), tetranucleotide (53 499, 0.58%), pentanucleotide (7355, 0.1%) and hexanucleotide (468, 0.01%) (Figure 1A). To facilitate retrieval from the database, SSRs were classified into two types based on their locations: the type I SSRs are located in genes and the type II SSRs are found in intergenic regions. The type I and type II account for 39.9% and 60.1% of the total SSRs, respectively, (Figure 1B). Depending upon the arrangement of nucleotides within the repeat motifs, four categories of SSRs can be defined (32, 33) (Figure 1C): (1) simple perfect SSRs (SP) with continuous repetitive units, e.g. (ATC)n; (2) simple imperfect SSRs (SIP) with one or more interruptions in the run of repeats, e.g. (ATC)mTT(ATC)n; (3) compound perfect SSRs (CP), typically, a mixture of SP SSRs, e.g. (AC)m(AGAC)n; (4) compound imperfect SSRs or interrupted CPs (CIP), a combination of compound and imperfect SSRs, e.g. (ATC)mTTG(AT)kGC(AT)n.

### Genome sequences and transcriptomic data

Wang *et al.* (20) provided the complete genome sequences of a female and a male grass carp. Three transcriptomic datasets were downloaded from the NCBI Sequence Read Archive (SRA) (34) (accession numbers: SRA099702, SRP041652 and SRP055685). The datasets in SRA format were converted to FASTQ format using the NCBI SRA Toolkit, then Q20 standard was applied to eliminate low-quality reads with a Perl script (IlluQC.pl) from NGSQCToolkit (35). The filtered transcriptomic reads were aligned against the grass carp genome using Burrows-Wheeler Aligner (BWA) (36) and were finally presented in JBrowse.

## Database features and utilizations

GCGD was constructed using the popular LAMP framework (Linux CentOS Server 7.1.1503, Apache 2.4.6, MySQL 5.5, and PHP 5.4.16). HTML5, CSS3 and JQuery were employed to optimize user experience. The website (http://bioinfo.ihb.ac.cn/gcgd) is supported by all major browsers. This database provides users with the facility to explore information such as genome annotation, gene function, transcriptome data and SSRs. All these data can be downloaded.

### Gene collinearity

Both the grass carp and the zebrafish belong to the family *Cyprinidae*, and they share 11 404 single-copy orthologous genes. Such a connection is presented in the ‘Gene Collinearity’ page. Considering that the assembly of the grass carp genome is still at a ‘draft’ stage and there are no counterparts for the 25 chromosomes of zebrafish, we picked up 40 scaffolds with most genes shared with zebrafish to illustrate their gene collinearity (Figure 1D). All collinear genes can be listed in a table by clicking on a chromosome or a scaffold.

### Gene family

A single gene might be duplicated to form a gene family on the genome. Generally genes within a gene family have similar biochemical functions, so researchers could save a lot of time in exploring a gene's function if they knew which gene family the gene belongs to. As described above, searching a gene results in a number of information including which gene family it belongs to. The ‘Gene Family’ page allows the user to browse all gene members for any gene family.

### Genetic map

The linkage maps are essential for mapping traits of interest and understanding genome evolution of the grass carp as a diploid species (2*n* = 48). All the 24 linkage groups of grass carp are displayed on the ‘Genetic Map’ page as an overview. Clicking on the chart of any specific linkage group brings a map in SVG (Scalable Vector Graphics) format with a vertical bar in the middle and marker names/genetic distance on either side (Figure 1E). The details of each marker can be shown by clicking on its name or genetic distance. A pull-down option frame is provided for switching among all the linkage groups.

### Gene search

**Figure 2.**
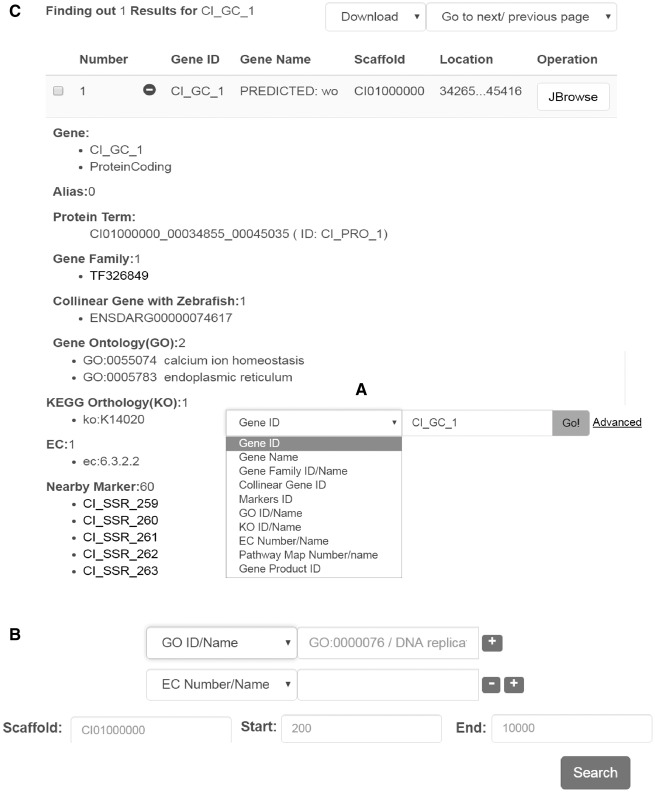
Interface for gene search and display. Data search can be performed in two different ways: (**A**) by keyword or under, (**B**) advanced search. (**C**) Example of the output generated upon looking for a particular gene. The results are displayed in a table format including the gene name/alias, location, sequences, and other functional features.

Genes can be searched simply by a keyword, gene name, EC number, or various other terms (Figure 2A). The ‘advanced’ hyperlink switches to a new searching page, in which predicted protein-coding genes can be screened by more criteria and by more gene annotations including EC terms, GO terms, KEGG terms, gene family information, collinear gene information (Figure 2B). Genes are displayed in a tabular form with fields including gene ID, gene name, scaffold and location (Figure 2C). If the ‘+’ icon in front of the gene ID is clicked on, more details are visible in an expanded space below the line (Figure 2C). Generally the details cover gene ID/name/alias, gene type, protein term, gene family, collinear genes with zebrafish, GO term, EC number, KEGG term, nearby markers, nucleic acid sequence and amino acid sequence.

### Genome visualization

**Figure 3.**
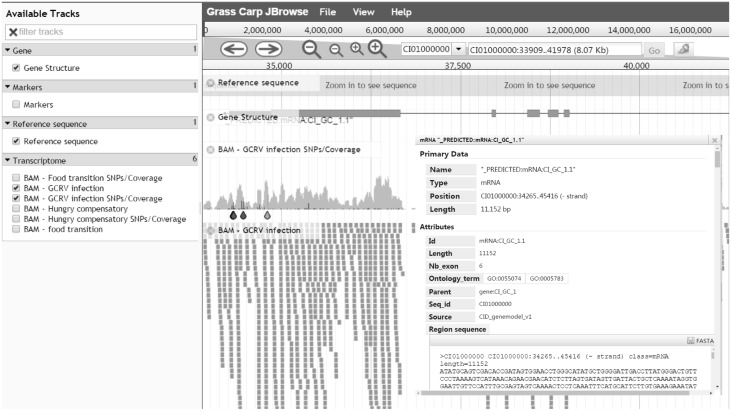
Genome visualization - JBrowse interface for navigation of genome annotations. All available data are displayed in the left frame for selection and selected data are graphically shown on the right frame. Details of a predicted protein-coding gene can be visualized after clicking on the gene.

When a search is performed, the resulting table contains a ‘JBrowse’ button which can be clicked to bring the user to an interactive genome browser (Figure 3). Biological features and dataset tracks are vertically listed on the left panel with check buttons for users to select. All checked features will be dynamically shown in JBrowse's main window. As an example, the detailed gene information only appears after clicking on the gene structure.

### SSR database

**Figure 4.**
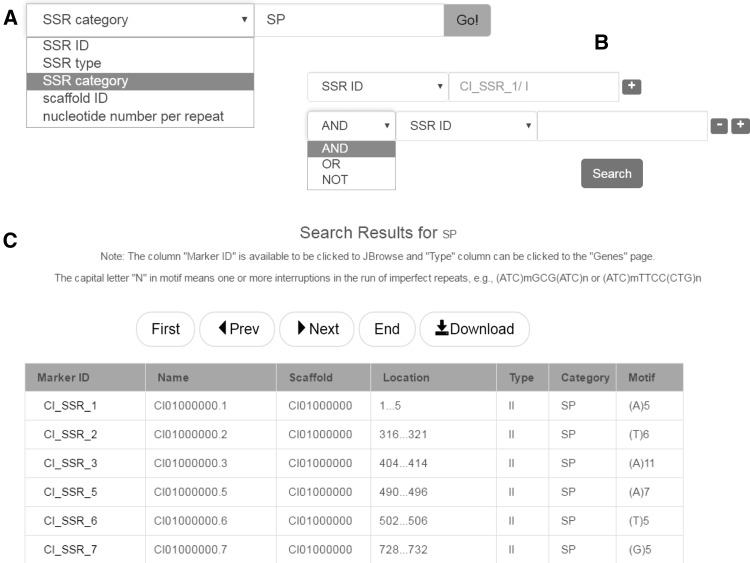
SSR database search. (**A**) Keyword search. (**B**) Advanced search. (**C**) The result of SSR database search.

SSRs are often referred to as microsatellites. Because of their advantages in the feature of codominance, high polymorphism and high repeatability, SSRs are broadly utilized for species or variety identification (37), DNA fingerprint profile identification (38), gene mapping and gene locating (39), linkage map construction (25) and molecular marker assisted breeding (36, 40). This database provides access to > 349 000 SSRs identified from the grass carp genome. The overall proportions of different types of SSR are shown as pie charts in the ‘Data statistics’ page. SSRs can be searched simply by a SSR term (Figure 4A), or any combination of these terms (Figure 4B). Search results are presented in tabular form (Figure 4C).

## Utilities for data mining

The NCBI Basic Local Alignment Search Tool (BLAST) program was integrated into GCGD web interface to allow users to facilitate sequence similarity search in grass carp and zebrafish. The operations include setting parameters and uploading query sequences in FASTA format, which can be pasted in a text box or be uploaded as a local file.

To assist the use of data, GCGD provided three other tools: IDConvert for term conversion, EC2KEGG for comparison of metabolic pathways and ReadContigs for sequence extraction. The disunity of terms in popular public databases hinders data exchange to a great extent, IDConvert enables the conversion of gene/protein IDs across GCGD and other databases including NCBI, Ensembl, ZFIN and Uniprot/Swiss-Prot. EC2KEGG is integrated for comparison of metabolic pathways between the grass carp and some other fishes or human. With a list of gene/protein IDs or EC numbers, EC2KEGG outputs the differences in every KEGG pathway map between the grass carp and the selected reference species. ReadContigs helps users to read segments from the draft genome of grass carp. With scaffold/contig names and sequence location information, requested sequences could be extracted and returned quickly.

## Summary and future development

Owing to the limited genetic and genomic data currently available, GCGD focuses on genome annotations, the visualization of omics data and the integration of tools for data mining. As a non-model fish, grass carp is far from being sufficiently studied on its functional genomics, and most annotations in GCGD were predicted by mapping with public databases. With the aid of a set of pipelines specially developed, the associations between GCGD terms and other databases were created. These associations can be conveniently updated as the field of the grass carp genomics develops or in relation to future databases of interest.

The long sexual maturity period of grass carp poses a huge challenge for traditional approaches for variety breeding, therefore molecular assisted breeding becomes inevitable. Currently, we integrated a database for microsatellites (SSRs), the widely used molecular markers in aquatic breeding. With increasing individual genomes sequenced, a SNP database within the framework of GCGD would be desirable, providing the most accurate information of these markers in the genome.

GCGD is expected to be a comprehensive resource hub and a workbench for genomics research in the grass carp, therefore continuous efforts are indispensable to expand data coverage, to add new content to data analysis, and to improve user experience. We are constantly collecting published omics data on the grass carp for GCGD. Oncoming datasets cover genome-wide investigations on DNA methylation, genome resequencing, various additional transcriptomes and lncRNA annotation. Meanwhile, novel tools under development are for GO/KEGG enrichment, variant calling, differential expression analysis and lncRNA prediction.

The gradation of a genome assembly may be accompanied by a number of changes in gene annotations. Since we are using a draft genome at this stage, caution is advised when using this database. According to our plan, each version of genome assembly will be coupled with a new version of GCGD, while the old versions will continue being accessible. This strategy is meaningful for those who want to generate analyses comparable to previous studies. Other features including the user interface are also subject to modification based on users' feedback.

## References

[1] Cudmore B. M.N.E. (2004) Biological Synopsis of Grass Carp (*Ctenopharyngodon idella*). Can. Manuscript Rep. Fish. Aquat. Sci., 7, 2705.

[2] Song X., Li S.F., Wang C.H. (2009) Grass carp (*Ctenopharyngodon idellus*) genetic structure analysis among native populations in china and introduced populations in USA, Europe and Japan based on mitochondrial sequence. Acta Hydrobiologica Sinica, 33, 709–716.

[3] Hu W., Chen J. (2015) Whole-genome sequencing opens a new era for molecular breeding of grass carp (*Ctenopharyngodon idellus*). Sci. China Life Sci., 58, 619–620. 25951934 10.1007/s11427-015-4864-x

[4] Sutton D.L., Vandiver V.V. (1986) Grass Carp: A Fish for Biological Management of Hydrilla and Other Aquatic Weeds in Florida. Agricultural Experiment Stations, Institute of Food and Agricultural Sciences, University of Florida.

[5] Huang R., Lv J., Luo D. (2012) Identification, characterization and the interaction of Tollip and IRAK-1 in grass carp (*Ctenopharyngodon idellus*). Fish Shellfish Immunol., 33, 459–467. 22659441 10.1016/j.fsi.2012.05.025

[6] Wang T.T., Song X.H., Bao G.M. (2013) Molecular characterization, expression analysis, and biological effects of interleukin-8 in grass carp *Ctenopharyngodon idellus*. Fish Shellfish Immunol., 35, 1421–1432. 23994423 10.1016/j.fsi.2013.08.006

[7] Leng X.J., Wu X.F., Tian J. (2012) Molecular cloning of fatty acid synthase from grass carp (*Ctenopharyngodon idella*) and the regulation of its expression by dietary fat level. Aquacult. Nutr., 18, 551–558.

[8] Yu E.M., Liu B.H., Wang G.J. (2014) Molecular cloning of type I collagen cDNA and nutritional regulation of type I collagen mRNA expression in grass carp. J. Anim. Physio. An. N., 98, 755–765. 10.1111/jpn.12132 24127725

[9] Liu L.W., Liang X.F., Li J. (2014) Feed intake, feed utilization and feeding-related gene expression response to dietary phytic acid for juvenile grass carp (*Ctenopharyngodon idellus*). Aquaculture, 424, 201–206.

[10] Li A.X., Yuan X.C., Liang X.F. (2016) Adaptations of lipid metabolism and food intake in response to low and high fat diets in juvenile grass carp (*Ctenopharyngodon idellus*). Aquaculture, 457, 43–49.

[11] Howe K., Clark M.D., Torroja C.F. (2013) The zebrafish reference genome sequence and its relationship to the human genome. Nature, 496, 498–503. 23594743 10.1038/nature12111 PMC3703927

[12] Xu P., Zhang X., Wang X. (2014) Genome sequence and genetic diversity of the common carp, *Cyprinus carpio*. Nat. Genet., 46, 1212–1219. 25240282 10.1038/ng.3098

[13] Kasahara M., Naruse K., Sasaki S. (2007) The medaka draft genome and insights into vertebrate genome evolution. Nature, 447, 714–719. 17554307 10.1038/nature05846

[14] Berthelot C., Brunet F., Chalopin D. (2014) The rainbow trout genome provides novel insights into evolution after whole-genome duplication in vertebrates. Nat. Commun., 5, 3657. 24755649 10.1038/ncomms4657 PMC4071752

[15] Lien S., Koop B.F., Sandve S.R. (2016) The Atlantic salmon genome provides insights into rediploidization. Nature, 533, 200. 27088604 10.1038/nature17164 PMC8127823

[16] Wu C., Zhang D., Kan M. (2014) The draft genome of the large yellow croaker reveals well-developed innate immunity. Nat. Commun., 5, 5227. 25407894 10.1038/ncomms6227 PMC4263168

[17] Sprague J., Clements D., Conlin T. (2003) The Zebrafish Information Network (ZFIN): the zebrafish model organism database. Nucleic Acids Res., 31, 241–243. 12519991 10.1093/nar/gkg027 PMC165474

[18] Howe D.G., Bradford Y.M., Conlin T. (2013) ZFIN, the Zebrafish Model Organism Database: increased support for mutants and transgenics. Nucleic Acids Res., 41, D854–D860. 23074187 10.1093/nar/gks938 PMC3531097

[19] Di Genova A., Aravena A., Zapata L. (2011) SalmonDB: a bioinformatics resource for Salmo salar and Oncorhynchus mykiss. Database (Oxford), 2011, bar050. 22120661 10.1093/database/bar050 PMC3225076

[20] Wang Y., Lu Y., Zhang Y. (2015) The draft genome of the grass carp (*Ctenopharyngodon idellus*) provides insights into its evolution and vegetarian adaptation. Nat. Genet., 47, 625–631. 25938946 10.1038/ng.3280

[21] Skinner M.E., Uzilov A.V., Stein L.D. (2009) JBrowse: a next-generation genome browser. Genome Res., 19, 1630–1638. 19570905 10.1101/gr.094607.109 PMC2752129

[22] McGinnis S., Madden T.L. (2004) BLAST: at the core of a powerful and diverse set of sequence analysis tools. Nucleic Acids Res., 32, W20–W25. 15215342 10.1093/nar/gkh435 PMC441573

[23] Porollo A. (2014) EC2KEGG: a command line tool for comparison of metabolic pathways. Source Code Biol. Med., 9, 1–4. 25202338 10.1186/1751-0473-9-19 PMC4157228

[24] Krzywinski M., Schein J., Birol I. (2009) Circos: an information aesthetic for comparative genomics. Genome Res., 19, 1639–1645. 19541911 10.1101/gr.092759.109 PMC2752132

[25] Xia J.H., Liu F., Zhu Z.Y. (2010) A consensus linkage map of the grass carp (*Ctenopharyngodon idella*) based on microsatellites and SNPs. BMC Genom., 11, 135. 10.1186/1471-2164-11-135 PMC2838847 20181260

[26] Voorrips R. (2002) MapChart: software for the graphical presentation of linkage maps and QTLs. J. Hered., 93, 77–78. 12011185 10.1093/jhered/93.1.77

[27] Conesa A., Gotz S., Garcia-Gomez J.M. (2005) Blast2GO: a universal tool for annotation, visualization and analysis in functional genomics research. Bioinformatics, 21, 3674–3676. 16081474 10.1093/bioinformatics/bti610

[28] Ashburner M., Ball C.A., Blake J.A. (2000) Gene ontology: tool for the unification of biology. The Gene Ontology Consortium. Nat. Genet., 25, 25–29. 10802651 10.1038/75556 PMC3037419

[29] Kanehisa M., Goto S. (2000) KEGG: kyoto encyclopedia of genes and genomes. Nucleic Acids Res., 28, 27–30. 10592173 10.1093/nar/28.1.27 PMC102409

[30] Schomburg I.,C.A., Schomburg D. (2002) BRENDA, enzyme data and metabolic information. Nucleic Acids Res., 30, 47–49. 11752250 10.1093/nar/30.1.47 PMC99121

[31] Li H., Coghlan A., Ruan J. (2006) TreeFam: a curated database of phylogenetic trees of animal gene families. Nucleic Acids Res., 34, D572–D580. 16381935 10.1093/nar/gkj118 PMC1347480

[32] Miah G., Rafii M.Y., Ismail M.R. (2013) A review of microsatellite markers and their applications in rice breeding programs to improve blast disease resistance. Int. J. Mol. Sci., 14, 22499–22528. 24240810 10.3390/ijms141122499 PMC3856076

[33] Wang M.L., Barkley N.A., Jenkins T.M. (2009) Microsatellite markers in plants and insects. Part I. Applications of biotechnology. Genes Genomes Genom., 3, 54–67.

[34] Leinonen R., Sugawara H., Shumway M. (2010) The sequence read archive. Nucleic Acids Res., 39, D19–D21. 21062823 10.1093/nar/gkq1019 PMC3013647

[35] Patel R.K., Jain M. (2012) NGS QC Toolkit: a toolkit for quality control of next generation sequencing data. PLoS One, 7, e30619. 22312429 10.1371/journal.pone.0030619 PMC3270013

[36] Ma H., Yang J., Su P. (2009) Genetic analysis of gynogenetic and common populations of Verasper moseri using SSR markers. Wuhan Univ. J. Nat. Sci., 14, 267–273.

[37] Briñez R., Caraballo O., Salazar V. (2011) Genetic diversity of six populations of red hybrid tilapia, using microsatellites genetic markers. Revista MVZ Córdoba, 16, 2491–2498.

[38] Chen Y., Dai X., Hou J. (2016) DNA fingerprinting of oil camellia cultivars with SSR markers. Tree Genet. Genomes, 12, 1–8.

[39] Chen X., Mei J., Wu J. (2015) A comprehensive transcriptome provides candidate genes for sex determination/differentiation and SSR/SNP markers in yellow catfish. Mar. Biotechnol., 17, 190–198. 25403497 10.1007/s10126-014-9607-7

[40] Jin S., Zhang X., Jia Z. (2012) Genetic linkage mapping and genetic analysis of QTL related to eye cross and eye diameter in common carp (*Cyprinus carpio L.*) using microsatellites and SNPs. Aquaculture, 358, 176–182. 10.4238/2015.April.17.5 25966124

